# Bilateral globus pallidus lesions associated with COVID-19: Mimicking acute carbon monoxide poisoning

**DOI:** 10.1097/MD.0000000000044183

**Published:** 2025-08-29

**Authors:** Jun Hyeon Park, Kyung Sik Yi, Chi-Hoon Choi, Yook Kim, Jisun Lee

**Affiliations:** a Department of Radiology, Chungbuk National University Hospital, Cheongju, Republic of Korea; b College of Medicine and Medical Research Institute, Chungbuk National University, Cheongju, Republic of Korea

**Keywords:** carbon monoxide, COVID-19-related encephalopathy, globus pallidus, MRI

## Abstract

**Rationale:**

Bilateral symmetrical globus pallidus (GP) involvement associated with coronavirus disease 2019 (COVID-19) infection is an extremely rare finding among cases of COVID-19-related encephalopathy.

**Patient concerns:**

A 76-year-old man was diagnosed with pneumonia and subsequently tested positive for COVID-19. He was transferred to the hospital on day 9 after symptom onset due to worsening respiratory distress and hypotension. Despite improvement in his pneumonia following the transfer, his mental status remained altered throughout his hospital stay.

**Diagnoses:**

A neurologic work-up, including brain magnetic resonance imaging (MRI) performed 2 weeks after the onset of altered mental state, revealed symmetrical high signal intensity lesions in the bilateral GP on MRI.

**Interventions:**

The patient received high-flow nasal oxygen therapy with norepinephrine infusion for hemodynamic support, along with intravenous meropenem and remdesivir.

**Outcomes:**

The patient gradually recovered and was able to engage in simple communication by the 3rd week. Follow-up MRI at 5 weeks showed resolution of GP lesions.

**Lessons:**

Differentiating COVID-19-related encephalopathy from acute carbon monoxide poisoning is challenging in the presence when bilateral symmetrical GP lesions are present. However, coexisting multiple ischemic lesions – common in COVID-19-related encephalopathy but rare in carbon monoxide poisoning – may serve as a useful radiologic clue.

## 1. Introduction

Coronavirus disease 2019 (COVID-19) presents with a wide range of clinical manifestations, from asymptomatic cases and mild respiratory symptoms such as fever and cough to severe disease leading to acute respiratory distress syndrome (ARDS).^[1]^ In addition to respiratory involvement, various neurological manifestations have been reported, including dizziness, headache, fatigue, dysgeusia, anosmia, impaired consciousness, acute cerebrovascular disease, ataxia, and seizures. Neurological symptoms are reported to occur in approximately 36% of patients with COVID-19.^[2]^ Although the global incidence of COVID-19 has significantly decreased at present, the potential for the emergence of new variants and future pandemics remains. Here, we report an extremely rare case of bilateral symmetric globus pallidus (GP) involvement in a patient with COVID-19 infection. This case highlights the importance of considering COVID-19-related encephalopathy in the differential diagnosis of bilateral GP lesions, which can mimic findings seen in acute carbon monoxide (CO) poisoning.

## 2. Case presentation

**Figure 1.**
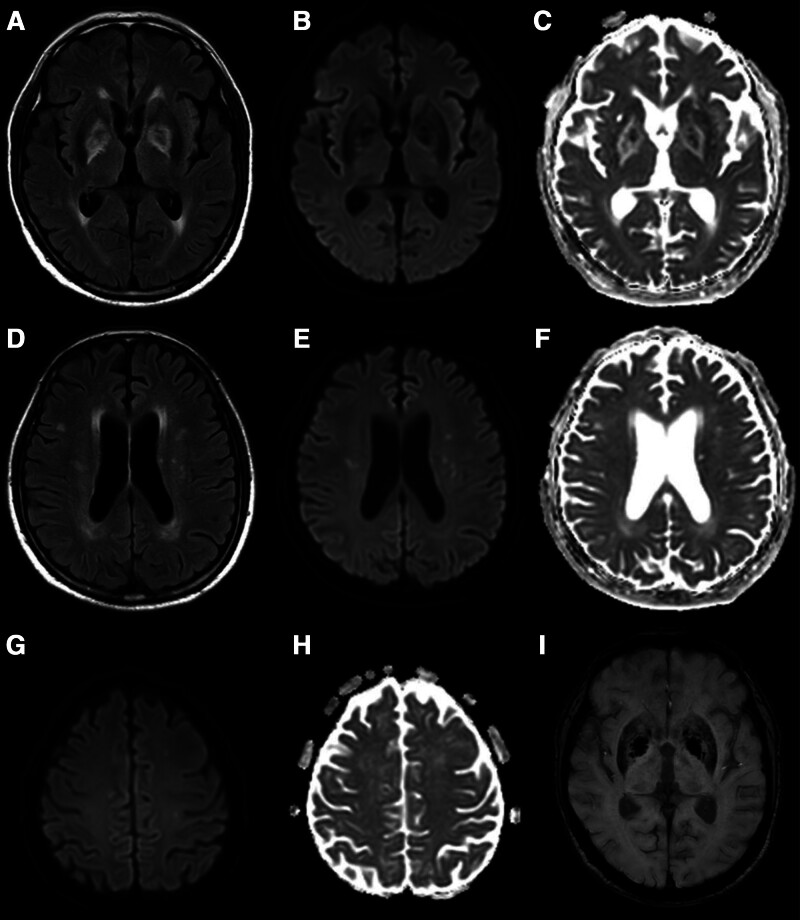
A 76-year-old man with COVID-19 and pneumonia was transferred to our hospital on day 9 after the onset of pneumonia symptoms due to worsening respiratory status and altered mental state. Because his drowsy mental state persisted, a brain MRI was performed 14 days after transfer. (A) The MRI revealed symmetrical high signal intensity lesions in the bilateral globus pallidus on a fluid attenuation inversion recovery image. (B, C) These lesions did not show restricted diffusion on DWI and the apparent diffusion coefficient map. (D–H) In addition, multiple punctate lesions with high DWI signal intensity were noted in the bilateral cerebral white matter. The distribution of these lesions did not correspond to typical watershed territories but was more consistent with an embolic infarction pattern. (I) There were no hemorrhagic changes observed in the globus pallidus on susceptibility-weighted imaging, apart from dark signal intensity due to iron deposition. COVID-19 = coronavirus disease 2019, DWI = diffusion-weighted imaging, MRI = magnetic resonance imaging.

**Figure 2.**
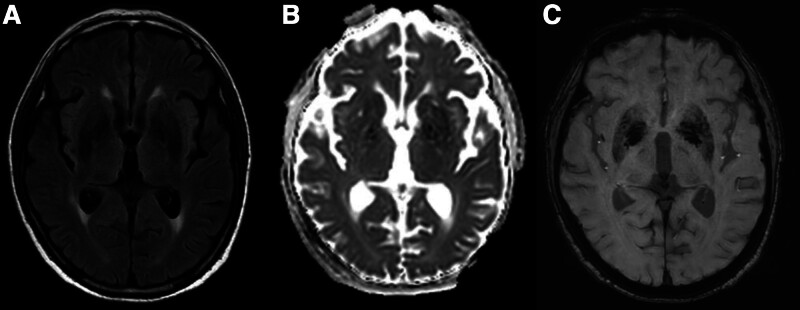
Follow-up brain MRI at 5 weeks after admission. (A–C) FLAIR, ADC, and SWI images demonstrate resolution of the previously noted bilateral globus pallidus lesions. ADC = apparent diffusion coefficient, FLAIR = fluid attenuation inversion recovery, SWI = susceptibility weighted imaging.

A 76-year-old man initially presented to an outside hospital with dyspnea and was treated for pneumonia. His past medical history included hypertension, diabetes mellitus, and asthma. While receiving treatment for pneumonia at the outside hospital, the patient’s respiratory distress worsened. On day 9 after symptom onset, the patient was diagnosed with COVID-19 by a positive reverse transcriptase polymerase chain reaction test using a nasopharyngeal swab specimen. He was subsequently transferred to our hospital for further management. At the time of transfer, he was on norepinephrine infusion at a dose of 0.1 μg/kg/min, and his blood pressure was 69/41 mm Hg. He was receiving oxygen therapy via high-flow nasal cannula (FiO_2_ 0.8, flow rate 50 L/min), with an oxygen saturation (SpO_2_) of 89%, and presented with an altered mental state. The patient was admitted to an isolation unit for intensive care and was treated with intravenous meropenem for pneumonia with abscess and remdesivir for COVID-19. Electrocardiogram monitoring showed normal sinus rhythm with ST and T wave abnormalities suggestive of inferior ischemia, while echocardiography revealed normal left ventricular systolic function without regional wall motion abnormality or right ventricular pressure overload. Although his pneumonia gradually improved during hospitalization, he remained in a drowsy mental state. After the isolation period ended, a neurologic work-up was initiated, and a brain magnetic resonance imaging (MRI) was performed on hospital day 14 following his transfer. The MRI revealed symmetrical high signal intensity lesions in the bilateral GP on fluid attenuation inversion recovery image (Fig. 1A). These lesions did not show restricted diffusion on diffusion-weighted imaging (Figs. 1B and C). In addition, multiple punctate fluid attenuation inversion recovery, diffusion-weighted imaging, and high signal intensity lesions were noted in the bilateral cerebral white matter (Fig. 1D–H). The distribution of these lesions did not correspond to typical watershed territories but was more consistent with an embolic infarction pattern. There were no hemorrhagic changes observed in the GP on susceptibility-weighted imaging, apart from dark signal intensity due to iron deposition (Figs. 1I and 2C). The patient had no history of CO exposure, and carboxyhemoglobin levels were not measured. In the context of the patient’s clinical course, the imaging findings were considered indicative of COVID-19-related neurologic involvement. The patient’s mental status gradually improved, and by the 3rd week after transfer to our hospital, he was able to engage in simple communication. A follow-up brain MRI performed 5 weeks after admission demonstrated resolution of the previously noted bilateral GP lesions (Fig. 2).

## 4. Discussion

COVID-19 is known to present not only with respiratory symptoms but also with various neurological manifestations. In a recently published systematic review, 3.4% of patients with COVID-19 who underwent neuroimaging showed abnormalities, including white matter abnormalities (17.6%), acute/subacute ischemic infarction (16.0%), encephalopathy (13.0%), and olfactory bulb abnormalities (23.1%).^[3]^ Such neurological involvement is more frequently reported in critically ill patients. The mechanisms underlying neurological complications of COVID-19 are thought to be multifactorial, including hypoxia, coagulopathy, cytokine storm, microvascular pathology, glial cell dysfunction, and direct central nervous system invasion.^[3–5]^ Among these complications, bilateral GP lesions are extremely rare, and only a few cases have been reported to date^[6–10]^ (Table 1). Notably, ARDS was present in all but 1 of these cases, suggesting a potential association between ARDS-related hypoxia and selective GP injury. In our case, while COVID-19 infection may have served as a precipitating factor, we believe that the bilateral GP lesions were not caused by the viral infection itself. Moreover, the imaging findings are not consistent with typical hypoxic–ischemic encephalopathy (HIE), as there was no involvement of brain regions commonly affected in hypoxic–ischemic encephalopathy – such as the cerebral cortex, thalami, hippocampi, or cerebellum. Instead, the selective, bilateral involvement of the GP supports a distinct pathophysiological mechanism likely involving a combination of systemic hypoxia, inflammation, and impaired perfusion associated with ARDS and shock. Nevertheless, a multifactorial etiology involving both ARDS-related hypoxia and COVID-19-related inflammatory responses cannot be definitively excluded.

**Table 1:** Summary of COVID-19 cases with bilateral globus pallidus involvement reported in the literature.

Authors	Age/sex	ARDS	Neurological manifestation	Brain lesions other than bilateral GP	Symptomatic improvement	Delayed progressive leukoencephalopathy
Ballout et al^[6]^	63/F	No	Rapidly progressive cognitive decline and psychomotor slowing	Corona radiata	Most neurological symptoms improved at 6-mo follow-up	Yes (follow-up MRI after 20 d)
Kulick-Soper et al^[7]^	52/F	Yes	Delayed consciousness recovery after sedation weaning	Hippocampus, substantia nigra, cerebral white matter, corpus callosum	–	–
Kurinobu et al^[8]^	27/M	Yes	Delayed consciousness recovery after sedation weaning	Substantia nigra, hippocampus	No improvement until hospital day 53; transferred to another hospital	No
Kurinobu et al^[8]^	61/F	Yes	Delayed consciousness recovery after sedation weaning	Substantia nigra, temporal lobe, subcortical frontal white matter, putamen	No improvement until hospital day 53; transferred to another hospital	–
Brun et al^[9]^	54/F	Yes	Delayed consciousness recovery after sedation weaning	Cerebral white matter	Consciousness recovered on day 10; right hemiplegia remained	No

ARDS = acute respiratory distress syndrome, COVID-19 = coronavirus disease 2019, F = female, GP = globus pallidus, M = male, MRI = magnetic resonance imaging.

Among various conditions associated with bilateral GP lesions, metabolic or toxic encephalopathy – particularly acute CO poisoning – is a well-known cause. In our case, although carboxyhemoglobin levels were not assessed, the clinical history strongly suggested that severe COVID-19 infection was the cause. Interestingly, GP lesions in COVID-19 share imaging features with CO poisoning, suggesting a similar underlying pathophysiology. Several mechanisms have been proposed for selective GP vulnerability in CO poisoning, including high metabolic activity, oxygen consumption, abundant mitochondrial content, rich vascular supply, and high iron concentration.^[11,12]^ Moreover, excessive catecholamine release, including dopamine, has been implicated as a cause of oxidative stress and neuronal injury in the GP.^[13]^ These mechanisms may also play a role in COVID-19-related GP lesions.

In addition, delayed neurologic sequelae (DNS) and progressive leukoencephalopathy are well-documented in CO poisoning, occurring in about 19.5% of patients, with white matter lesions observed in approximately 30% of those with DNS.^[14]^ Although DNS was not observed in our case, progressive leukoencephalopathy following COVID-19-related GP lesions has been reported in at least 1 case,^[6]^ suggesting that progressive demyelination may occur via a shared neuroinflammatory pathway.^[15]^ Therefore, careful monitoring for delayed demyelinating processes should be considered in similar cases.

For differentiation between COVID-19-related GP lesions and those caused by CO poisoning, a comprehensive assessment of clinical history, carboxyhemoglobin levels, and radiologic features is essential. Notably, as seen in our case and other reports (Table 1), multiple ischemic lesions presumed to be embolic infarctions often accompany GP lesions in COVID-19 infection. In contrast, ischemic stroke is relatively uncommon in CO poisoning,^[16]^ suggesting that the presence of such ischemic lesions may serve as an important radiologic clue for differential diagnosis.

This case has a limitation. Although COVID-19 was confirmed by a positive reverse transcriptase polymerase chain reaction test using a nasopharyngeal swab specimen, cerebrospinal fluid analysis – including testing for severe acute respiratory syndrome coronavirus 2 antibodies – was not performed. Therefore, direct central nervous system involvement by the virus could not be evaluated, and the exact etiology still remains unclear.

## 5. Conclusion

We report an extremely rare case of bilateral symmetrical GP lesions as a neurological manifestation of COVID-19. These lesions share imaging features with acute CO poisoning, and neuroinflammation may contribute to their pathogenesis. However, coexisting multiple ischemic lesions, which commonly seen in COVID-19 but uncommon in CO poisoning may serve as a useful radiologic clue for differentiation between these conditions. Given the potential for DNS, careful clinical and radiologic follow-up is recommended.

## Author contributions

**Conceptualization:** Jun Hyeon Park, Kyung Sik Yi.

**Data curation:** Jun Hyeon Park, Kyung Sik Yi.

**Writing – original draft:** Jun Hyeon Park, Kyung Sik Yi.

**Writing – review and editing:** Jun Hyeon Park, Kyung Sik Yi, Chi-Hoon Choi, Yook Kim, Jisun Lee.

**Supervision:** Kyung Sik Yi, Chi-Hoon Choi, Yook Kim, Jisun Lee.
